# Long- and short-term fluctuations compared for several organ systems across sleep stages

**DOI:** 10.3389/fnetp.2022.937130

**Published:** 2022-09-09

**Authors:** Johannes Zschocke, Ronny P. Bartsch, Martin Glos, Thomas Penzel, Rafael Mikolajczyk, Jan W. Kantelhardt

**Affiliations:** ^1^ Institute of Medical Epidemiology, Biometrics and Informatics (IMEBI), Interdisciplinary Center for Health Sciences, Martin-Luther-University Halle-Wittenberg, Halle, Germany; ^2^ Institute of Physics, Martin-Luther-University Halle-Wittenberg, Halle, Germany; ^3^ Department of Physics, Bar-Ilan University, Ramat Gan, Israel; ^4^ Interdisciplinary Sleep Medicine Center, Charité - Universitätsmedizin Berlin, Berlin, Germany

**Keywords:** time series analysis, long-term correlations, persistence, scaling analysis, heartbeat, pulse-transit time, respiration, brain-wave amplitudes

## Abstract

Some details of cardiovascular and cardio-respiratory regulation and their changes during different sleep stages remain still unknown. In this paper we compared the fluctuations of heart rate, pulse rate, respiration frequency, and pulse transit times as well as EEG alpha-band power on time scales from 6 to 200 s during different sleep stages in order to better understand regulatory pathways. The five considered time series were derived from ECG, photoplethysmogram, nasal air flow, and central electrode EEG measurements from full-night polysomnography recordings of 246 subjects with suspected sleep disorders. We applied detrended fluctuation analysis, distinguishing between short-term (6–16 s) and long-term (50–200 s) correlations, i.e., scaling behavior characterized by the fluctuation exponents *α*_1_ and *α*_2_ related with parasympathetic and sympathetic control, respectively. While heart rate (and pulse rate) are characterized by sex and age-dependent short-term correlations, their long-term correlations exhibit the well-known sleep stage dependence: weak long-term correlations during non-REM sleep and pronounced long-term correlations during REM sleep and wakefulness. In contrast, pulse transit times, which are believed to be mainly affected by blood pressure and arterial stiffness, do not show differences between short-term and long-term exponents. This is in constrast to previous results for blood pressure time series, where *α*_1_ was much larger than *α*_2_, and therefore questions a very close relation between pulse transit times and blood pressure values. Nevertheless, very similar sleep-stage dependent differences are observed for the long-term fluctuation exponent *α*_2_ in all considered signals including EEG alpha-band power. In conclusion, we found that the observed fluctuation exponents are very robust and hardly modified by body mass index, alcohol consumption, smoking, or sleep disorders. The long-term fluctuations of all observed systems seem to be modulated by patterns following sleep stages generated in the brain and thus regulated in a similar manner, while short-term regulations differ between the organ systems. Deviations from the reported dependence in any of the signals should be indicative of problems in the function of the particular organ system or its control mechanisms.

## 1 Introduction

The regulation of quasi-periodic processes in the human body is characterized by high degree of complexity. Therefore, fluctuations in physiological signals often show nonlinear dynamics and correlation behavior with (fractal) scaling relations (Bassingthwaighte and Raymond, 1994; West, 2014). For example, a scaling behavior of the power spectra similar to 1/*f* ^*β*^ (“coloured”) noise has been observed in series of time intervals between successive heartbeats, breaths, and steps (Kobayashi and Musha, 1982; Peng et al., 1993b; Hausdorff et al., 1995; Peng et al., 2002; Ivanov et al., 2009). Their dynamics are modified by different physiological states (e.g., sleep/wake, sleep stages) and activities, aging, and under pathological conditions (Ivanov et al., 1999b; Bunde et al., 2000; Hausdorff et al., 2001; Karasik et al., 2002; Goldberger et al., 2002; Kantelhardt et al., 2003; Bartsch et al., 2007). Coloured noise is equivalent to long-term correlations as described by a slowly decaying autocorrelation function (“persistence”) (Bunde et al., 2000; Kantelhardt, 2011). Short-term correlations, on the other hand, are equivalent to exponentially (i.e., rather quickly) decaying autocorrelations and characterized by white noise fluctuations at low frequencies.

By comparing the correlation behavior of many physiological signals across different states in many subjects, hypotheses regarding the control mechanisms for the underlying physiological systems can be derived. Here, we studied, for the first time, the dynamics of pulse-transit times, from heart to finger, in a similar way as previously done for inter-heartbeat intervals (Peng et al., 1993a; Bunde et al., 2000; Schumann et al., 2010), respiratory intervals (Kantelhardt et al., 2003; Schumann et al., 2010) and brain-wave amplitudes (Kantelhardt et al., 2015). In particular, the dynamics of the control of the pulse-wave propagation—e.g., blood pressure, arterial stiffness, etc.—can potentially be studied (Guo et al., 2022). Changes of the scaling behavior in some subjects can also be used as early indicators or diagnostic tools for pathologies that affect one or many of the studied organ systems (Ivanov et al., 1999a; Goldberger et al., 2002).

Specifically, in this paper based on polysomnography (PSG) recordings from a clinical sleep laboratory, we studied the short- and long-term correlations (“persistence”) in five time series characterizing different organ systems.

• **RRI** (R-R intervals; heart): The time intervals between successive R peaks in the electrocardiogram (ECG) as an expression of autonomic cardiac control.
• **PPI** (pulse to pulse intervals; cardiovascular system): The time intervals between successive pulse wave peaks derived from a photoplethysmogram (PPG) as an expression of autonomic cardiac control but slightly influenced by pulse wave velocity regulation mechanisms.
• **PTT** (pulse transit times, cardiovascular system): The time intervals between each R peak (in the ECG) and the corresponding pulse wave peak (in the PPG), believed to be an expression of blood pressure (Allen and Kyriacou, 2022) and arterial stiffness.
• **BBI** (breath to breath intervals; respiratory system): The time intervals between successive respiration maxima during the sleep phase as an expression of autonomic respiratory control.
• **EEG** (brain): The alpha-band amplitudes of a centrally recorded electroencephalogram (EEG, electrodes C3 or C4) as an expression of brain dynamics.

Based on previous work in the field, we address the following hypotheses for short-term (*α*_1_) and long-term (*α*_2_) fluctuation exponents, calculated for these five time series and probably related to parasympathetic and sympathetic control, respectively. Our implied medical hypotheses are that deviations from normal dependence should be indicative of problems in the function or control mechanisms of the particular organ system.

1) Short-term correlations (*α*_1_) for RRI do slightly depend on sleep stages and have a maximum for intermediate age groups (Schumann et al., 2010).
2) Long-term correlations (*α*_2_) for RRI are weaker than short-term correlations and nearly absent during non-REM sleep (N2 and N3), but pronounced during wakefulness and REM sleep (Bunde et al., 2000; Schumann et al., 2010).
3) The scaling behavior of RRI and PPI is very similar. This is expected because the two time series are closely linked (Schäfer and Vagedes, 2013).
4) The *α*_2_ scaling behavior of BBI is similar to RRI, but the BBI correlations are generally weaker, particularly during wakefulness and REM sleep (Kantelhardt et al., 2003; Schumann et al., 2010). Different trends of *α*_2_ with aging occur for RRI and BBI in REM sleep and wakefulness (Schumann et al., 2010).
5) There is no relevant influence of respiratory disorders—in particular, sleep apnea as indicated by the apnea-hypopnea index (AHI)—on the long-term scaling behavior of RRI and BBI (Penzel et al., 2003).
6) Average PTTs decrease with aging due to increasing arterial stiffness (Nichols, 2005).

In addition, studying the fluctuation scaling behavior in PTTs for the first time, we address the following novel hypotheses:

7) Long-term correlations (*α*_2_) for PTT are similar to BBI.
8) Short-term correlations (*α*_1_) for PTT are weaker than for any of the other considered time series, there is hardly any crossover (i.e., *α*_1_ = *α*_2_), and *α*_1_ is only weakly changing with age. This hypothesis implies that there is no close relationship between PTT and blood pressure at short time scales, since very strong (even non-stationary, *α*_1_ > 1) short-term correlations have previously been reported for blood pressure time series (Galhardo et al., 2009; Fuchs et al., 2010; Castiglioni et al., 2020).
9) An increased body mass index (BMI) is associated with increased PTT short-term correlations (*α*_1_) during N2 sleep. Increased alcohol consumption is associated with decreased PTT short-term correlations (*α*_1_) during nocturnal wakefulness but not during sleep.

## 2 Methods

### 2.1 Data recordings

We analyzed single night PSG data from 246 subjects with suspected sleep disorders recorded in clinical sleep laboratories at the Charité-Universitätsmedizin Berlin, Germany, between April 2017 and March 2019. The study was approved by the ethics committee of the Charité-Universitätsmedizin Berlin and registered at the German Clinical Trial Register (DRKS) with ID DRKS00016908. All enrolled subjects gave written informed consent prior to the study. Full PSG including EEG, electrooculogram (EOG), electromyogram (EMG), ECG, PPG, oxygen saturation, and respiratory effort was recorded using either an Embla^®^ (Natus, Pleasanton, United States) or a SOMNOscreen™ PSG system (SOMNOmedics, Randersacker, Germany). The final used data set consisted of single-night recordings of 130 female and 116 male participants with body mass index 28.3 ± 6.2 (17.0, 51.9) kg/m^2^, age 51.2 ± 14.2 (18, 79.6) years, and time in bed 7.5 ± 0.8 (2.3,7.9) hours [mean ± standard deviation (minimum, maximum)].

All recordings were part of diagnostic examination and were classified by the current rules of the International Classification of Sleep Disorders (ICSD-3); multiple diagnoses are possible. The dataset includes 12 subjects without sleep disorders, 132 subjects with sleep-related breathing disorders, 70 subjects with insomnia, 32 subjects with central disorders of hypersomnolence, 37 subjects with sleep-related movement disorder, 8 subjects with parasomnias and 8 subjects with circadian rhythm sleep-wake disorders.

### 2.2 Data preprocessing

Each measurement was cropped to only contain data between the ‘lights off’ and ‘lights on’ time stamps, indicating beginning and end of the sleep opportunity period, respectively. Sleep stages based on 30-second epochs have been determined from the PSG data by trained experts following standard guidelines of the American Academy of Sleep Medicine (AASM) (Berry et al., 2018) to distinguish light sleep (stages N1 and N2), deep sleep (stage N3), and rapid eye movement (REM) sleep. We disregarded the N1 sleep episodes, since they were too short for the time series analysis in most subjects, hence distinguishing nocturnal wakefulness, N2, N3, and REM sleep.

Heartbeats were detected as R peaks in the ECGs using the Biosppy algorithm (Carreiras et al., 2015). Intervals between successive R peaks (RRIs) were regarded as normal if 1) RRI_*i*_ > 330 ms, 2) RRI_*i*_ < 2000 ms, and 3) 0.7 RRI_*i*−1_ < RRI_*i*_ < 1.6 RRI_*i*−1_. Non-normal RRIs were discarded and the remaining data segments stitched together. Chen et al. (2002) and Ma et al. (2010) have shown that cutting out and stitching together data segments obtained from discontinuous experimental recordings does not affect the outcome of the Detrended Fluctuation Analysis (DFA, see Section 2.3). From the normal RRIs, we calculated the average heartbeat interval, the standard deviation of normal-to-normal intervals (SDNN), and the standard deviations of the RRI increments (i.e., the root mean sum of squared distance, RMSSD) applying standard heart rate variability (HRV) analysis (Malik, 1996).

Pulse wave peaks were extracted from the PPGs using the intersecting tangents method (Hemon and Phillips, 2016). This approach determines the intersection between the tangent of the PPG slopes maximum and the (horizontal) tangent of its minimum, yielding the point of pulse arrival at the finger tip. We have also considered other definitions of pulse wave peaks, such as maxima or minima of the PPG signal, but—as in Hemon and Phillips, (2016)—the intersecting tangents method showed the best correspondence with R peaks. The reason behind this observation lies in the changes of pulse wave shape with aging, since the systolic peak of the pulse wave gets broader with increasing age, and therefore more inaccurate in comparison to heart beats (Kelly et al., 1989). We have also applied an offset correction (subtracting a moving average over 3 s) and a low pass filter [moving average over 0.1 s (Hemon and Phillips, 2016)] to the raw PPG signal. In order to calculate the tangent at the point of maximal slope, we used the first derivative of the PPG signal, which was high pass filtered again (moving average over 0.1 s), to reduce noise. Intervals between successive pulse wave peaks (PPIs) were regarded as normal within the same limits as for RRIs. Again, non-normal PPIs were discarded and the remaining data segments stitched together, and averages as well as statistics corresponding to SDNN and RMSSD were calculated.

Pulse transit times (PTTs) were defined as time differences between a detected R peak and the corresponding pulse wave peak at the finger. Specifically, the pulse wave peak had to occur between 0.1 and 0.8 s after the R peak. Due to missing pulse wave peaks (temporarily low quality PPG signal, etc.) not every R peak could be matched with a corresponding pulse wave peak. All successfully derived segments of PTT series (0.1 s < PTT_*i*_ < 0.8 s) were stitched together, and averages as well as standard deviations and standard deviations of the increments were calculated.

Respiratory cycles were detected in the respiratory flow signal by identification of the maxima. The signal was preprocessed by subtracting a 10 s moving average and applying a high pass filter (1 s moving average); see Leube et al. (2020) for details. We have also considered other methods to derive respiratory intervals, but—as we focused on respiration cycles rather than the true respiration onset—the maxima methods turned out to be the most robust approach. Intervals between successive respiration peaks (BBIs) were regarded as normal if 1) BBI_*i*_ > 2 s, 2) BBI _*i*_ < 8 s, and 3) 0.7 BBI_*i*−1_ < BBI_*i*_ < 1.6 BBI_*i*−1_. Non-normal BBIs were discarded and the remaining data segments stitched together.

Brain-wave amplitudes for the alpha-band were derived from the C4 (or C3) electrode EEG recordings by 1) employing the Fourier filtering technique (Theiler et al., 1992) to extract the alpha-band oscillations in the range from 7 to 12 Hz, 2) applying a Hilbert transform to determine the instantaneous amplitudes for each sampling point of the recording, and 3) re-sampling to one amplitude value per second (rate 1 Hz). For a detailed description of the procedure, we refer to Kantelhardt et al. (2015).

### 2.3 Detrended fluctuation analysis to characterize correlation behavior

In the final step of our analysis procedure, for each subject, we split all time series according to sleep stages (wakefulness, N2, N3, REM sleep), applied DFA with second order polynomial detrending (DFA2), and averaged the fluctuation functions for each stage with statistical weights corresponding to the duration of each episode. The DFA method first introduced by Peng et al. (1994) for studying DNA sequences has been intensely applied to study persistence (auto-correlations) in noisy, non-stationary time series and later been improved for higher-order detrending (Bunde et al., 2000). The method quantifies fluctuations on different time scales *s*, see Kantelhardt et al. (2001) for details. In brief, for each *s* the integrated (cumulated) signal of length *N* is split into non-overlapping pieces (segments) of length *s*. Within each segment an *n*-th order polynomial fit is subtracted, and the remaining mean-square fluctuations are averaged. Repeating the procedure for many scales *s* yields the square of the DFA function *F*(*s*), which corresponds to a detrended standard derivation on many time scales *s*.

In case of long-term (power-law) correlated data without trends, the scaling behavior of the fluctuation function, *F*(*s*) ∼ *s* ^*α*^ with scaling exponent *α* > 0.5, is equivalent to a scaling of the signal’s power spectrum, *P*(*f*) ∼ *f* ^−*β*^ with frequency *f* and *β* = 2*α* − 1 (Bartsch et al., 2005). If the data is stationary, i.e., *α* < 1 and *β* < 1, this is also equivalent to a scaling of the autocorrelation function *C*(*s*) ∼ *s* ^−*γ*^ with *γ* = 2(1 − *α*) = 1 − *β* (Bashan et al., 2008). The advantage of using DFA and studying *F*(*s*) instead of *P*(*f*) or *C*(*s*) lies mainly in the detrending capability, that allows analyzing nonstationary data. For data with only short-term correlations, the scaling exponents approach *α* = 0.5 and *β* = 0 for asymptotically large *s* and small *f*, respectively. By determining the effective scaling exponents *α*_1_ and *α*_2_ for small and large scales, respectively, we can distinguish the scaling behavior of short- and long-term fluctuations.

**FIGURE 1:**
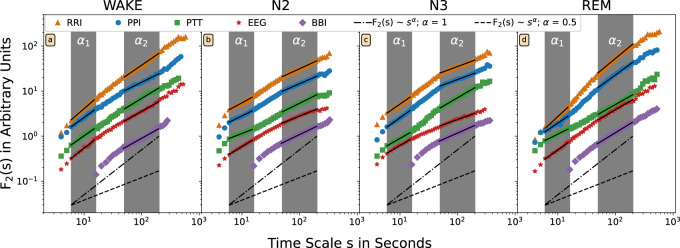
The DFA2 fluctuation functions *F*(*s*) are shown on double-logarithmic plot for one exemplary subject and different nocturnal states **(A)** wakefulness, **(B)** N2 (light) sleep, **(C)** N3 (deep) sleep, and **(D)** REM sleep. Time intervals between successive R peaks in the ECG (orange, triangles), between successive pulse waves peaks (blue, circles), for pulse wave transition times (green, squares), for respiratory intervals (violet, rhombuses), and for alpha-band amplitudes in the EEG (red, stars) have been analyzed. The scale on the horizontal axis has been rescaled by a factor of four for the respiratory data. The dashed lines with slope α = 0.5 (random white noise) and the dash-dotted lines with slope 1.0 (1/*f*-type correlated noise) are shown for comparison. The two fitting regimes for the effective short-term fluctuation exponent *α*_1_ (6–16 heartbeat intervals or seconds) and the effective long-term fluctuation exponent *α*_2_ (50–200 heartbeat intervals or seconds; 12 to 50 breaths) are marked by the gray shading. The fluctuation functions have been shifted vertically for better visualization.

Figure 1 shows such DFA functions on a double-logarithmic plot for an exemplary subject, the four different nocturnal sleep stages, and all five time series as described in the Introduction. In addition, we shaded in gray the areas for determining short-term scaling exponents *α*_1_ (from 6 to 16 s) and for long-term scaling exponents *α*_2_ (from 50 to 200 s). A scaling exponent *α* is, by definition, the linear slope of the fluctuation function in the double-logarithmic plot. In Figure 1 the corresponding linear fits for *α*_1_ and *α*_2_ are plotted as black lines.

Short-term and long-term scaling exponents were calculated for each subject, each signal, and each stage. However, to ensure the data quality, in further analysis only scaling exponents with coefficient of determination *r* ^2^ > 0.9 were included. Since the total durations of the sleep stages differ, we report the results for each stage separately and did not calculate weighted averages over the entire sleep period.

## 3 Results

**FIGURE 2:**
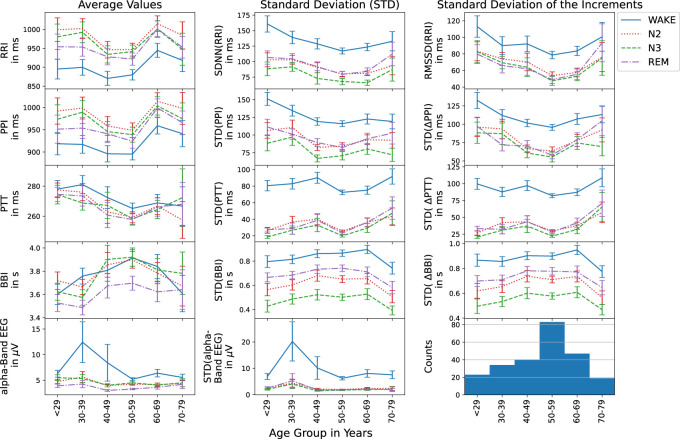
Average values (first column), standard deviations (second column), and standard deviations of the increments (third column) are shown versus age group for RRI (top row), PPI (second row), PTT (third row), BBI (forth row), and EEG (bottom row) during different nocturnal states (see legend on the right hand side). The histogram in the bottom right corner presents the numbers of subjects for each age group. Error bars indicate the standard error.

The following results are averages of each considered quantity for the whole group of subjects, often divided into 10-year age groups^1^; see the bottom right histogram in Figure 2 for the age distribution in our sample. In all cases, the four nocturnal states, wakefulness, N2, N3, and REM sleep have been studied separately.

### 3.1 Age dependencies of averages and variabilities

First, we studied the average values, the standard deviations (corresponding to the HRV parameter SDNN for heartbeat intervals) and standard deviations of the increments (corresponding to the HRV parameter RMSSD for heartbeat intervals) for all five time series, RRI, PPI, PTT, BBI and EEG (see Introduction) to check if they follow the physiological expectations. Figure 2 shows these three standard parameters versus the age groups. As Schmitt et al. (2009) have already shown for RRI and two age groups, there is an age dependence as well as differences between the sleep stages. As expected, average RRIs were shortest during wake, while at the same time, SDNN and RMSSD showed the largest values. The parameters during N2 and REM were always very similar. For N3 (deep sleep), however, slightly lower values of SDNN were observed. For all three statistical parameters, minimum values occurred around the 50–59 years old group in all stages. As also expected (see Hypothesis 3 in the Introduction), exactly the same behavior of the statistical parameters was observed for PPI, since RRI and PPI are closely related during rest and sleep as already reported by Schäfer and Vagedes, (2013).

The study of PTTs in the third row of Figure 2 yielded the expected behavior that generally average PTTs decrease with age (see Hypothesis 6). This occurred for all nocturnal stages, although slight deviations for the first and last age group cannot be excluded within the error bars (standard error). Standard deviations of PTT and standard deviation of PTT increments showed similarly small values during all three sleep stages, but much larger values (by a factor of approximately three) during nocturnal wakefulness. The generally increasing but somewhat non-monotonous trend with increasing age was identical for both standard deviation parameters and all nocturnal states.

The breathing intervals exhibited the most pronounced differences between N2, N3 and REM sleep. While average BBI were shortest during REM sleep and of similar length for N2 and N3 sleep, the two standard deviation parameters were smallest during N3, followed by N2 and REM as expected. Wakefulness yielded the largest BBI standard deviations. Each standard deviation parameter showed the same age dependence for all sleep stages. However, for the average respiration period, BBI, a slightly different age dependence was observed during wakefulness.

EEG alpha-band brain wave amplitudes, also showed the expected behavior with clearly much larger values during wakefulness as compared to sleep. These differences seem to become slightly weaker with increasing age.

### 3.2 Age dependences of short- and long-term correlations

**FIGURE 3:**
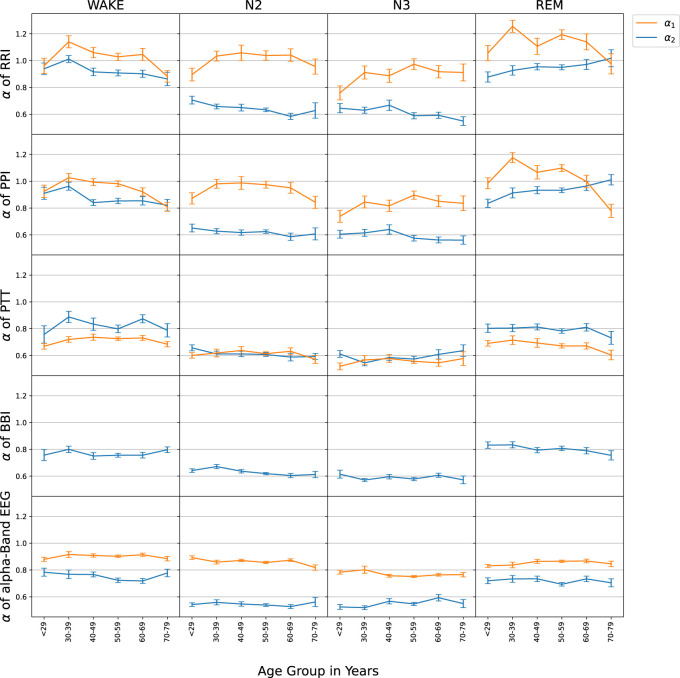
The age-dependence of short-term (*α*_1_, orange) and long-term (*α*_2_, blue) scaling exponents is shown for the series of time intervals between successive R peaks in the ECG (RRI, first row), time intervals between successive pulse wave peaks at the finger tip (PPI, second row), pulse transit times (PTT, third row), respiratory intervals (BBI, fourth row), and EEG amplitudes in the alpha-band (EEG, bottom row). For the evaluation, nocturnal wake states (left column), N2 sleep (second column), N3 sleep (third column) and REM sleep (right column) were separated. All subjects were binned to 10-year age groups; the standard error of each point for each group is indicated by the vertical error bars.

Figure 3 summarizes the results of the DFA2 fluctuation scaling analysis for the five considered time series during different sleep stages. The results for short-term correlations of RRI (*α*_1_) in the first row confirmed our Hypothesis 1, although the maximum for intermediate age groups was a bit broader than in Schumann et al. (2010) and reached the largest values at lower ages ( ≈35 instead of ≈55 years). The long-term correlations of RRI (*α*_2_) in the first row together with the results for brain-wave amplitudes in the bottom row clearly confirmed our Hypothesis 2. Since the results for PPI (second row) were—within the error bars—identical with those for RRI (first row), our Hypothesis 3 was also confirmed.

The results regarding BBI were also in agreement with Schumann et al. (2010). Since we observed decreasing (increasing) *α*_2_ exponents with aging for RRI and PPI during wakefulness (REM sleep), but slightly decreasing *α*_2_ exponents with aging for BBI during REM sleep, Hypothesis 4 was also confirmed. Note however, that the observed age dependence of BBI during wakefulness was not the same as in Schumann et al. (2010).

Our main findings for PTT confirmed hypotheses (7), long-term correlations (*α*_2_) for PTT were similar to BBI and (8), short-term correlations (*α*_1_) were weaker than for any of the other considered time series. It is important to note that for PTT particularly during non-REM sleep (N2 and N3) short and long-term correlations became identical (*α*_1_ = *α*_2_), so that the crossover disappeared. Furthermore, there was no change of PTT *α*_1_ with aging. Such behavior was not observed for any of the other signals. PTT and EEG data did not seem to have a relevant age dependence, except for a possible slight decay of PTT short-term correlations (*α*_1_) with age above 40 years during wakefulness and REM sleep.

### 3.3 Influences of sex, BMI, smoking, alcohol consumption, and sleep apnea

**FIGURE 4:**
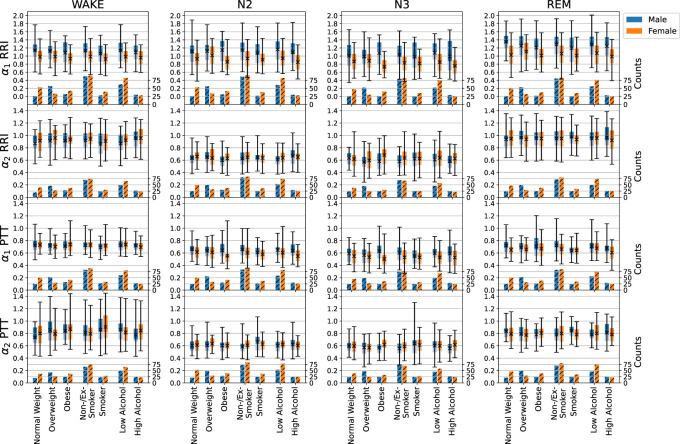
Influences of sex (male—blue, female—orange), BMI, smoking, and alcohol consumption on short- and long-term fluctuation exponents *α*_1_ and *α*_2_ of RRI and PTT, indicated by the boxplots. The light part of each box represents the values between the lower quartile and the median, and the dark part represents the values between the median and the upper quartile. The ends of the whiskers mark the 2.5% quantile and the 97.5% quantile, respectively. The total average values appear as black crosses in the box plot (see scale on the left axis for the boxplots). Additionally, numbers of subjects available for each analysis are shown in the hatched bars on the bottom of each plot (scale on the right axis). In particular, we compared normal weight (BMI < 25 kg/m^2^) versus overweight (BMI 25–30 kg/m^2^) and obese (BMI > 30 kg/m^2^), non-/ex-smokers versus smokers, and subjects with low alcohol consumption ( ≤ once per month) versus subjects with high alcohol consumption ( > once per month).

Figure 4 shows how the short- and long-term scaling exponents for RRI and PTT depend on sex, BMI, smoking status and alcohol consumption. Interestingly, we found a strong (highly significant, *p* < 0.001) sex dependence of the short-term (*α*_1_) correlations in RRI (as well as those in PPI), which were consistently higher in males than in females across all sleep stages. In contrast, the long-term (*α*_2_) correlations in RRI were not sex depended. For short- and long-term correlation in PTT only marginal differences between males and females were seen, with some significance reached for *α*_1_ during N2, N3, and REM sleep (*p* = 0.020, 0.008, 0.032, respectively).

An increasing BMI led to slightly increasing short-term correlations (*α*_1_) in PTT but not in RRI or any of the other considered signals. Multivariable regression analysis^2^, adjusted for age (in categories of 10 years as shown before) and sex, showed a significant increase of *α*_1_ for PTT with BMI during N2 sleep (*p* = 0.010). The same trend was observed for breathing intervals (BBI, not shown). Body size was associated with short-term correlations (*α*_1_) of the heart (also not shown). However, this effect was mainly due to the increase of male population with increasing body size and therefore not relevant.

Smoking led to a slightly higher heart rate (not shown), but hardly affected SDNN and RMSSD over all sleep stages. In the multivariable regression analysis, short-term correlations (*α*_1_) were significantly decreased for smokers during wakefulness regarding RRI (*p* = 0.038) and during REM sleep regarding PTT (*p* = 0.012), while long-term correlations (*α*_2_) for PTT were significantly increased for smokers during wakefulness (*p* = 0.007) and N3 (*p* = 0.021) sleep (see also Figure 4).

While the mean values of RRI and PPI increased (slower heartbeat) with habitual alcohol consumption, PTT, BBI and EEG amplitudes stayed rather constant. We also saw a decline of PTT and EEG averages and possibly an increase in the average BBI associated with a large increase of BBI standard deviations. Regarding the short- and long-term correlations, only a slight decline of *α*_1_ (short-term correlations) for RRI during N3 sleep (*p* = 0.048) and for PTT with increasing alcohol consumption was significant during wakefulness (*p* = 0.029).

Among the many possible disorders, our sample is most suitable for addressing the effects of sleep apnea. This sleep-related breathing disorder can be classified by the apnea hypopnea index (AHI), which is defined as the average number of apneas and hypopneas per hour of sleep. We distinguished healthy subjects (AHI < 5/h), mild apnea (5–15/h), moderate apnea (15–30/h), and severe apnea ( > 30/h). While the mean values of RRI, PPI, and PTT clearly decreased with increasing AHI during all four considered states, their standard deviations were clearly decreasing only during wakefulness. For BBI, we observed an increase of the standard deviation with AHI as expected, since the apneas represent a stopping of respiration. Mean respiratory intervals seemed to peak for the moderate apnea group. Regarding the short- and long-term correlations, however, we did not observe any relevant changes with increasing AHI. This finding is consistent with Penzel et al. (2003) and confirmed our Hypothesis 5. A detailed analysis of five-minute epochs before apneas, after apneas, and far from apneas did not reveal any significant differences in the short- and long-term fluctuation exponents, even if we studied central apneas, obstructive apneas, mixed apneas and hypopneas separately.

Furthermore, we did not see any significant effect of the ICSD-3 classifications on the scaling behavior of RRI, PPI, PTT, BBI, and EEG. This could be due to the small numbers of patients in some of the subgroups. In particular, we had only 12 subjects without sleep related disease in our control group. As sleep disorders (sleep-related breathing disorders and insomnia) can have several causes and show very large variability, a systematic manifestation in the examined signals (heart rate, pulse transit time, respiration and EEG) is not visible in our method of analysis.

## 4 Discussion

Our results in Figure 2 showed that the average values, standard deviations and standard deviations of increments for RRI, PPI, PTT, and BBI depend on sleep stages, while some of them change non-linearly with aging. Regarding RRI, i.e., for HRV parameters, similar changes have previously been reported by Schmitt et al. (2009) for young and elderly subjects. For 24 h averages, Umetani et al. (1998) reported that the HRV parameters SDNN and RMSSD decreased till the age of 50–60 years and then stabilized. This is consistent with our observations. Other studies reported decreases of heart rate and HRV parameters with aging (Voss et al., 2012; Xhyheri et al., 2012; Jensen-Urstad et al., 1997). Since a decreasing heart rate corresponds to increasing average RRI values, these observations are not really coinciding with ours, see Figure 2 top left panel. A reason could be that we focused on subjects with sleep related disorders instead of healthy subjects. Hence, a study with better statistics and a focus on healthy subjects as well as sleep stage distinction is needed for a full clarification.

Furthermore, we found a close similarity between HRV parameters (from RRI, top row in Figure 2) and pulse rate variability parameters (from PPI, second row in Figure 2), confirming our Hypothesis 3 from the Introduction. Chen et al. (2015) and Khandoker et al. (2011) have reported differences between these two kinds of parameters during apnea events, but no differences during normal breathing. Compared with our work, they studied shorter episodes (2–3 min) of apnea or non-apnea data, while we averaged over all identical sleep stages for the whole night, so that the influence of apneas is probably averaged out in our results. We note that Constant et al. (1999) have shown that pulse rate variability can solely be induced by respiratory modulations as in their study on children with a fixed cardiac pace maker rhythm, possible effects of heart rate variability on PPI have been excluded.

To our knowledge, this is the first paper that analyzes long nocturnal series of PTT values, defined by the time intervals between R peaks and corresponding pulse wave peaks at the finger. We confirmed Hypothesis 6 based on Nichols, (2005) that average PTT decreases with aging, likely due to increasing arterial stiffness. There was hardly any sleep-stage dependence of the average PTT values. However, like HRV (i.e., SDNN and RMSSD), the two PTT variability parameters clearly decreased during sleep as compared to wakefulness (see Figure 2 center and right panels). Hardly any further decay in the PTT variabilities occurred from REM to N2 to N3 sleep, although respiratory variability clearly followed this decaying order. No clear age dependence could be observed for PTT or BBI variabilities. More statistics and a focus on healthy subjects is needed for a full clarification of these dependencies.

The results for the correlation behavior on short and long time scales, i.e., the exponents *α*_1_ and *α*_2_ of RRI, PPI, PTT, BBI, and EEG alpha-band amplitude data were presented in Figure 3. Our results regarding RRI and BBI are fully consistent with those reported in previous work (Bunde et al., 2000; Kantelhardt et al., 2003; Schumann et al., 2008; Schumann et al., 2010). In particular, short-term correlations (*α*_1_) for RRI did only weakly depend on sleep stages and had a maximum for intermediate age groups, confirming our Hypothesis 1. This suggests that the short-term autonomic control system of the heart is not strongly affected by sleep-stage related brain activity and that it ages in a non-monotonous way. Further research is needed to clarify the reasons behind this unusual age dependence.

Long-term correlations (*α*_2_) for RRI were weaker than short-term correlations and nearly absent during non-REM sleep (N2 and N3), but pronounced during wakefulness and REM sleep, confirming our Hypothesis 2. This pattern, reflecting the scaling behavior of EEG alpha-band amplitudes, indicates that the long-term autonomic control system of the heart is significantly affected by cerebral activity via sympathetic control, or, alternatively, both are driven by the same regulatory process (Günther et al., 2022). Since the type of long-term correlations strongly differs between non-REM sleep on the one hand and REM sleep or wakefulness on the other hand, this dependence strongly indicates that an influence from the brain is involved, because sleep stages originate in the brain. In our opinion, it is not plausible that an organ or organ system would independently from the brain create correlations that simultaneously change with those in brain dynamics following exactly the same sleep-stage stratification pattern. Again, we observed a very similar behavior for PPIs (cp. first and second row in Figure 3), also confirming our Hypothesis 3 from the Introduction (Schäfer and Vagedes, 2013).

Nevertheless, a comparison of the results for males and females in Figure 4 revealed significant differences, which had not been observed in previous studies of healthy subjects (Schumann et al., 2008; Schumann et al., 2010). In particular, *α*_1_ values were higher in men than in women, while *α*_2_ values were similar. This finding may be related with differences in parasympathetic control in men and women. However, since most of our subjects had some kind of sleep-related disorder (see end of Section 2.1), we cannot exclude an effect of these disorders on our results, and suggest that a scaling analysis of data from a larger group of healthy subjects is needed for a clarification. Possibly, HRV parameters reported to be higher in men than in women (Umetani et al., 1998) may be related with this observation.

Our medical hypothesis is that organ-specific alternations in the long-term fluctuation pattern (which seems to originate in the brain) or short-term fluctuation pattern (with a more local origin) can indicate medical problems related with this organ. For example, if short-term fluctuations of RRI are described by a lower exponent *α*_1_ than expected for the age of the subject, this could be a hint towards premature aging of the cardiovascular system. Or if long-term RRI fluctuations follow a nearly random behavior (low *α*_2_) not only during non-REM sleep, but also during REM sleep and/or wakefulness, this could indicate a diminished sympathetic input. On the other hand, a high *α*_2_ also during non-REM sleep could indicate insufficiency of the cardiovascular system to relax, which in turn may negatively affect sleep quality. However, since we do not have data from subjects with specific cardiac problems or diagnoses, such hypotheses cannot be tested in this study.

Comparing the long-term (*α*_2_) scaling behaviors of RRI and BBI, we confirmed previous reports of a very similar sleep-stage dependence, but somewhat weaker correlations in BBI, particularly during wakefulness and REM sleep (Kantelhardt et al., 2003; Schumann et al., 2010) (Figure 3), indicating that cerebral activity also influences respiration during sleep. Different trends of *α*_2_ with aging occurred for RRI and BBI in wakefulness and REM sleep, confirming our Hypothesis 4. This suggests that aging affects autonomic cardiac and respiratory control in somewhat different ways. While our results regarding RRI were fully in agreement with Schumann et al. (2010), the trends for aging were less clear for BBI, so that a study with more data and of healthy subjects would be needed for a clarification of this detail.

Our results for the nonlinear dynamics of PTT series on short and long time scales, i.e., their exponents *α*_1_ and *α*_2_, are novel and thus cannot be directly compared with previously published results. Surprisingly, we found that there were no differences between the short- and long-term fluctuation scaling behavior (i.e., *α*_1_ = *α*_2_) during non-REM (N2 and N3) sleep, so that no crossover occurs, see Figure 3. This might indicate that only one control process is relevant for PTT during non-REM sleep, and no additional short-term correlations are introduced into PTT. An alternative interpretation is that the parasympathetic and sympathetic control of PTT are well and identically balanced during the different sleep and wake stages so that practically no crossover occurrs. During wakefulness and REM sleep, slight differences between *α*_1_ and *α*_2_ were observed, but—contrary to the behavior of RRI, PPI and EEG amplitude data—*α*_1_ was *smaller* than *α*_2_ for all age groups.

According to a standard textbook (Allen and Kyriacou, 2022) PTT intervals are mainly affected by blood pressure, although varying levels of arterial stiffness and body and limb positions do also play a certain role. Since body and limb positions do not often change during the sleep phase, their influence does probably not yield a relevant contribution to the observed PTT fluctuations. Moreover, the stroke volume mainly affects the pulse wave amplitude (which we do not study here), but not the timing. Regarding arterial stiffness, we are not aware of specific studies that address their short- and long-term fluctuation behavior, so that currently no conclusions regarding its (sleep-stage or age-dependent) influence on PTT seems possible. Blood pressure, on the other hand, is known to be strongly correlated on short time scales with *α*_1_ values of 1.4 for mice (Galhardo et al., 2009) and 1.2 for humans (Fuchs et al., 2010) during wakefulness, increasing to 1.3–1.4 during the night (Castiglioni et al., 2020). We are not aware of published data regarding differences between the sleep stages. Hence, if the fluctuations of PTT intervals would mainly reflect blood pressure changes, a similarly large *α*_1_ value would have to occur for PTT, which is not the case. Therefore, our result seems to indicate that PTT is only reflecting long-term fluctuations of blood pressure, while short-term correlations of PTT must be dominated by faster and much closer to random fluctuations of arterial stiffness. It thus suggests that the parasympathetic control of short-term variations in arterial stiffness has no short-term memory (variations close to random) and is not directly linked to autonomic cardiac and respiratory control.

The sleep stage dependence of *α*_2_ for PTT is very similar to BBI. This suggests that long-term PTT fluctuations are similarly controlled via the sympathetic nervous system as long-term respiratory fluctuations and also linked to cerebral activity. We did not observe pronounced changes of PTT scaling behavior with aging and only a marginally significant sex dependence, see Figure 3 (third row) and Figure 4 (third and fourth row).

An increased body mass index (BMI) was associated with slightly increased PTT short-term correlations (*α*_1_) during N2 sleep. Increased alcohol consumption was associated with decreased PTT short-term correlations (*α*_1_) during nocturnal wakefulness but not during sleep. For smokers, short-term correlations (*α*_1_) in RRI decreased during wakefulness, while those in PTT decreased during REM sleep; long-term correlations (*α*_2_) in PTT increased during wakefulness and N3 sleep. We think that a study with more subjects is needed to confirm these apparently not very systematic effects, before a medical interpretation can be provided. Nevertheless, the observation of changes for PTT but (in most cases) not for the other considered signals suggests that PTTs yield independent information, probably related with changes in arterial stiffness control and should be included in subsequent work.

Consistent with previous reports [see, e.g., Penzel et al. (2003)] we did not observe any relevant changes of the scaling behaviors of either RRI, PPI, PTT, BBI, or EEG alpha-band amplitudes with increasing disease severity of apnea (i. e., AHI) nor with other sleep disorders. This suggests that the observed scaling behaviors of these signals and their long-term autonomic control are very robust. However, studies with a larger samples are needed for clarification.

Limitations of our study include our sample size of just 246 subjects, mainly with sleep related disorders, while most previous studies regarding temporal correlations in biosignals and HRV focused on healthy subjects.

## 5 Summary and conclusion

We confirmed the sleep-stage and age dependence of basic statistical parameters characterizing cardiovascular, respiratory, and brain dynamics, including mean RRI and its deviations (SDNN, RMSSD), mean PPI and its deviations, mean PTT and its deviations, mean BBI and its deviations and mean EEG alpha-band amplitude and its deviation during different sleep stages. Additionally, we investigated systematically aspects of nonlinear dynamics and the correlation behavior of these time series by calculating the DFA exponents *α*_1_ and *α*_2_. While the long-term correlations (*α*_2_) of all analyzed physiological systems follow the same sleep-stage stratification pattern, indicating a common regulatory mechanism, short-term correlations do hardly vary across sleep stages and may be governed by organ-specific physiological processes. Surprisingly, PTT is an exception from this rule, since we have observed a complete absence of additional short-term fluctuations, i.e., *α*_1_ ≈ *α*_2_ across all age groups and sleep stages. This result indicates that short-term PTT fluctuations do not reflect short-term blood pressure fluctuations, which are rather characterized by very different exponents *α*_1_ > 1.

## Data Availability

The data are available from the project “Long- and short-term fluctuations compared for several organ systems across sleep stages” at OSFHOME (osf.io; location Frankfurt, Germany) under the doi 10.17605/OSF.IO/3R4PU.

## Footnotes

1 The age group 29 years contains two subjects below 20 years; both are 18 years old.

2 Multivariable regression analysis was done in python using the package *statsmodels* from Seabold and Perktold, (2010).

## References

[B1] Allen J. Kyriacou P. (2022). Photoplethysmography. Academic Press.

[B2] Bartsch R. Hennig T. Heinen A. Heinrichs S. Maass P. (2005). Statistical analysis of fluctuations in the ECG morphology. Phys. A 354, 415–431. 10.1016/j.physa.2005.03.019

[B3] Bartsch R. Plotnik M. Kantelhardt J. W. Havlin S. Giladi N. Hausdorff J. M. (2007). Fluctuation and synchronization of gait intervals and gait force profiles distinguish stages of Parkinson’s disease. Phys. A 383, 455–465. 10.1016/j.physa.2007.04.120 PMC2156195 18163154

[B4] Bashan A. Bartsch R. Kantelhardt J. W. Havlin S. (2008). Comparison of detrending methods for fluctuation analysis. Phys. A 387, 5080–5090. 10.1016/j.physa.2008.04.023

[B5] Bassingthwaighte J. B. Raymond G. M. (1994). Evaluating rescaled ranged analysis for time series. Ann. Biomed. Eng. 22, 432–444. 10.1007/BF02368250 7998689

[B6] Berry R. B. Albertario C. L. Harding S. M. Lloyd R. M. Plante D. T. Quan S. F. (2018). Darien, IL. The AASM manual for the scoring of sleep and associated events: Rules, terminology and technical specifications, version 2.5. Am. Acad. Sleep Med.

[B7] Bunde A. Havlin S. Kantelhardt J. W. Penzel T. Peter J. H. Voigt K. (2000). Correlated and uncorrelated regions in heart-rate fluctuations during sleep. Phys. Rev. Lett. 85, 3736–3739. 10.1103/PhysRevLett.85.3736 11030994

[B8] Carreiras C. Lourenço A. Canento F. Silva H. Fred A. (2015). Biosppy: Biosignal processing in python. Available at: https://github.com/PIA-Group/BioSPPy/ (Accessed March, 2022).

[B9] Castiglioni P. Omboni S. Parati G. Faini A. (2020). Day and night changes of cardiovascular complexity: A multi-fractal multi-scale analysis. Entropy 22, 462. 10.3390/e22040462 33286236 PMC7516947

[B10] Chen Z. Ivanov P. C. Hu K. Stanley H. E. (2002). Effect of nonstationarities on detrended fluctuation analysis. Phys. Rev. E 65, 041107. 10.1103/PhysRevE.65.041107 12005806

[B11] Chen X. Huang Y.-Y. Yun F. Chen T.-J. Li J. (2015). Effect of changes in sympathovagal balance on the accuracy of heart rate variability obtained from photoplethysmography. Exp. Ther. Med. 10, 2311–2318. 10.3892/etm.2015.2784 26668634 PMC4665692

[B12] Constant I. Laude D. Murat I. Elghozi J.-L. (1999). Pulse rate variability is not a surrogate for heart rate variability. Clin. Sci. 97, 391. 10.1042/CS19990062 10491338

[B13] Fuchs K. Schumann A. Y. Kuhnhold A. Guzik P. Piskorski J. Schmidt G. (2010). “Comparing analysis of heart rate and blood pressure fluctuations in healthy subjects,” in Proc. 6th Conference of the European Study Group on Cardiovascular Oscillations, Berlin, Germany.

[B14] Galhardo C. E. C. Penna T. J. P. Argollo de Menezes M. Soares P. P. S. (2009). Detrended fluctuation analysis of a systolic blood pressure control loop. New J. Phys. 11, 103005. 10.1088/1367-2630/11/10/103005

[B15] Goldberger A. L. Amaral L. A. N. Hausdorff J. M. Ivanov P. C. Peng C.-K. Stanley H. E. (2002). Fractal dynamics in physiology: Alterations with disease and aging. Proc. Natl. Acad. Sci. U. S. A. 99 (1), 2466–2472. 10.1073/pnas.012579499 11875196 PMC128562

[B16] Günther M. Kantelhardt J. W. Bartsch R. P. (2022). The reconstruction of causal networks in physiology. Front. Netw. Physiol. 2. 10.3389/fnetp.2022.893743 PMC10013035 36926108

[B17] Guo C. Jiang Z. He H. Liao Y. Zhang D. (2022). Wrist pulse signal acquisition and analysis for disease diagnosis: A review. Comput. Biol. Med. 143, 105312. 10.1016/j.compbiomed.2022.105312 35203039

[B18] Hausdorff J. M. Peng C.-K. Ladin Z. Wei J. Y. Goldberger A. L. (1995). Is walking a random walk? Evidence for long-range correlations in stride interval of human gait. J. Appl. Physiol. 78, 349–358. 10.1152/jappl.1995.78.1.349 7713836

[B19] Hausdorff J. M. Ashkenazy Y. Peng C.-K. Ivanov P. C. Stanley H. E. Goldberger A. L. (2001). When human walking becomes random walking: Fractal analysis and modeling of gait rhythm fluctuations. Phys. A 302, 138–147. 10.1016/s0378-4371(01)00460-5 12033228

[B20] Hemon M. C. Phillips J. P. (2016). Comparison of foot finding methods for deriving instantaneous pulse rates from photoplethysmographic signals. J. Clin. Monit. Comput. 30, 157–168. 10.1007/s10877-015-9695-6 25902897

[B21] Ivanov P. C. Amaral L. A. Goldberger A. L. Havlin S. Rosenblum M. G. Struzik Z. R. (1999a). Multifractality in human heartbeat dynamics. Nature 399, 461–465. 10.1038/20924 10365957

[B22] Ivanov P. C. Bunde A. Amaral L. N. Havlin S. Fritsch-Yelle J. Baevsky R. M. (1999b). Sleep-wake differences in scaling behavior of the human heartbeat: Analysis of terrestrial and long-term space flight data. Europhys. Lett. 48, 594–600. 10.1209/epl/i1999-00525-0 11542917

[B23] Ivanov P. C. Ma Q. D. Bartsch R. P. Hausdorff J. M. Amaral L. A. N. Schulte-Frohlinde V. (2009). Levels of complexity in scale-invariant neural signals. Phys. Rev. E 79, 041920. 10.1103/PhysRevE.79.041920 PMC6653582 19518269

[B24] Jensen-Urstad K. Storck N. Bouvier F. Ericson M. Lindblad L. E. Jensen-Urstad M. (1997). Heart rate variability in healthy subjects is related to age and gender. Acta Physiol. Scand. 160, 235–241. 10.1046/j.1365-201X.1997.00142.x 9246386

[B25] Kantelhardt J. W. Koscielny-Bunde E. Rego H. H. Havlin S. Bunde A. (2001). Detecting long-range correlations with detrended fluctuation analysis. Phys. A 295, 441–454. 10.1016/S0378-4371(01)00144-3

[B26] Kantelhardt J. W. Penzel T. Rostig S. Becker H. F. Havlin S. Bunde A. (2003). Breathing during rem and non-rem sleep: Correlated versus uncorrelated behaviour. Phys. A 319, 447–457. 10.1016/S0378-4371(02)01502-9

[B27] Kantelhardt J. W. Tismer S. Gans F. Schumann A. Y. Penzel T. (2015). Scaling behavior of eeg amplitude and frequency time series across sleep stages. EPL Europhys. Lett. 112, 18001. 10.1209/0295-5075/112/18001

[B28] Kantelhardt J. W. (2011). “Fractal and multifractal time series,” in Mathematics of complexity and dynamical systems. Editor Meyers R. (New York, NY: Springer). 10.1007/978-1-4614-1806-1_30

[B29] Karasik R. Sapir N. Ashkenazy Y. Ivanov P. C. Dvir I. Lavie P. (2002). Correlation differences in heartbeat fluctuations during rest and exercise. Phys. Rev. E 66, 062902. 10.1103/PhysRevE.66.062902 12513330

[B30] Kelly R. Hayward C. Avolio A. O’Rourke M. (1989). Noninvasive determination of age-related changes in the human arterial pulse. Circulation 80, 1652–1659. 10.1161/01.cir.80.6.1652 2598428

[B31] Khandoker A. H. Karmakar C. K. Palaniswami M. (2011). Comparison of pulse rate variability with heart rate variability during obstructive sleep apnea. Med. Eng. Phys. 33, 204–209. 10.1016/j.medengphy.2010.09.020 20980188

[B32] Kobayashi M. Musha T. (1982). 1/f fluctuation of heartbeat period. IEEE Trans. Biomed. Eng. 29, 456–457. 10.1109/TBME.1982.324972 7106796

[B33] Leube J. Zschocke J. Kluge M. Pelikan L. Graf A. Glos M. (2020). Reconstruction of the respiratory signal through ecg and wrist accelerometer data. Sci. Rep. 10, 14530. 10.1038/s41598-020-71539-0 32884062 PMC7471298

[B34] Ma Q. D. Bartsch R. P. Bernaola-Galván P. Yoneyama M. Ivanov P. C. (2010). Effect of extreme data loss on long-range correlated and anticorrelated signals quantified by detrended fluctuation analysis. Phys. Rev. E 81, 031101. 10.1103/PhysRevE.81.031101 PMC3534784 20365691

[B35] Malik M. (1996). Heart rate variability. Ann. Noninvasive Electrocardiol. 1, 151–181. 10.1111/j.1542-474X.1996.tb00275.x PMC6931824 29938866

[B36] Nichols W. W. (2005). Clinical measurement of arterial stiffness obtained from noninvasive pressure waveforms. Am. J. Hypertens. 18, 3S–10S. 10.1016/j.amjhyper.2004.10.009 15683725

[B37] Peng C.-K. Buldyrev S. V. Goldberger A. L. Havlin S. Simons M. Stanley H. E. (1993a). Finite-size effects on long-range correlations: Implications for analyzing dna sequences. Phys. Rev. E 47, 3730–3733. 10.1103/PhysRevE.47.3730 9960430

[B38] Peng C.-K. Mietus J. Hausdorff J. Havlin S. Stanley H. E. Goldberger A. L. (1993b). Long-range anticorrelations and non-Gaussian behavior of the heartbeat. Phys. Rev. Lett. 70, 1343–1346. 10.1103/PhysRevLett.70.1343 10054352

[B39] Peng C. K. Buldyrev S. V. Havlin S. Simons M. Stanley H. E. Goldberger A. L. (1994). Mosaic organization of dna nucleotides. Phys. Rev. E 49, 1685–1689. 10.1103/PhysRevE.49.1685 9961383

[B40] Peng C.-K. Mietus J. E. Liu Y. Lee C. Hausdorff J. M. Stanley H. E. (2002). Quantifying fractal dynamics of human respiration: Age and gender effects. Ann. Biomed. Eng. 30, 683–692. 10.1114/1.1481053 12108842

[B41] Penzel T. Kantelhardt J. W. Grote L. Peter J.-H. Bunde A. (2003). Comparison of detrended fluctuation analysis and spectral analysis for heart rate variability in sleep and sleep apnea. IEEE Trans. Biomed. Eng. 50, 1143–1151. 10.1109/TBME.2003.817636 14560767

[B42] Schäfer A. Vagedes J. (2013). How accurate is pulse rate variability as an estimate of heart rate variability? A review on studies comparing photoplethysmographic technology with an electrocardiogram. Int. J. Cardiol. 166, 15–29. 10.1016/j.ijcard.2012.03.119 22809539

[B43] Schmitt D. T. Stein P. K. Ivanov P. C. (2009). Stratification pattern of static and scale-invariant dynamic measures of heartbeat fluctuations across sleep stages in young and elderly. IEEE Trans. Biomed. Eng. 56, 1564–1573. 10.1109/TBME.2009.2014819 19203874 PMC2821156

[B44] Schumann A. Y. Bauer A. Penzel T. Schmidt G. Kantelhardt J. W. (2008). “Cardiovascular oscillations and correlations during sleep,” in Proc. 5th Conference of the European Study Group on Cardiovascular Oscillations (Parma Italy).

[B45] Schumann A. Y. Bartsch R. P. Penzel T. Ivanov P. C. Kantelhardt J. W. (2010). Aging effects on cardiac and respiratory dynamics in healthy subjects across sleep stages. Sleep 33, 943–955. 10.1093/sleep/33.7.943 20614854 PMC2894436

[B46] Seabold S. Perktold J. (2010). Statsmodels: Econometric and statistical modeling with python. Proc. Python Sci. Conf., 92–96. 10.25080/Majora-92bf1922-011

[B47] Theiler J. Eubank S. Longtin A. Galdrikian B. Farmer J. D. (1992). Testing for nonlinearity in time series: The method of surrogate data. Phys. D. Nonlinear Phenom. 58, 77–94. 10.1016/0167-2789(92)90102-S

[B48] Umetani K. Singer D. H. McCraty R. Atkinson M. (1998). Twenty-four hour time domain heart rate variability and heart rate: Relations to age and gender over nine decades. J. Am. Coll. Cardiol. 31, 593–601. 10.1016/S0735-1097(97)00554-8 9502641

[B49] Voss A. Heitmann A. Schroeder R. Peters A. Perz S. (2012). Short-term heart rate variability–age dependence in healthy subjects. Physiol. Meas. 33, 1289–1311. 10.1088/0967-3334/33/8/1289 22813869

[B50] West B. J. (2014). A mathematics for medicine: The network effect. Front. Physiol. 5, 456. 10.3389/fphys.2014.00456 25538622 PMC4260484

[B51] Xhyheri B. Manfrini O. Mazzolini M. Pizzi C. Bugiardini R. (2012). Heart rate variability today. Prog. Cardiovasc. Dis. 55, 321–331. 10.1016/j.pcad.2012.09.001 23217437

